# Genome Sequences of 408 SARS-CoV-2 Strains Obtained from Nasopharyngeal Swabs in La Araucanía Region, Southern Chile

**DOI:** 10.1128/mra.00121-22

**Published:** 2022-03-28

**Authors:** Michel Abanto Marin, Benjamín Leyton-Carcaman, Claudio Vázquez, Benjamín Durán-Vinet, Katterin Bobadilla, Claudio Rodriguez-Ruiz, Andrés San Martín, Pía Riquelme, Fernando Baez, María José Carrasco, Gloria Rodríguez-Moretti, Álvaro Cerda, Nicolás Saavedra, María de la Luz Mora

**Affiliations:** a Genomics and Bioinformatics Unit, Scientific and Technological Bioresource Nucleus (BIOREN), Universidad de La Frontera, Temuco, Chile; b Innovation and Entrepreneurship Institute (iDEAUFRO), Universidad de La Frontera, Temuco, Chile; c Subdepartamento de Epidemiología, Departamento de Salud Pública y Planificación Sanitaria, Seremi de Salud Región de La Araucanía, Temuco, Chile; d Subdepartamento Laboratorio Clínico, Hospital Dr. Hernán Henríquez Aravena de Temuco, Temuco, Chile; e Department of Basic Sciences, Center of Excellence in Translational Medicine, Scientific and Technological Bioresource Nucleus (BIOREN), Universidad de La Frontera, Temuco, Chile; f Center of Molecular Biology & Pharmacogenetics, Scientific and Technological Bioresource Nucleus (BIOREN), Universidad de La Frontera, Temuco, Chile; Queens College CUNY

## Abstract

Here, we announce the genome sequences of 408 strains of severe acute respiratory syndrome coronavirus 2 (SARS-CoV-2) obtained from nasopharyngeal swabs in the Araucanía Region, Southern Chile. The genomes obtained are valuable to expand the availability of useful genomic data for future epidemiological studies of SARS-CoV-2 in Chile and worldwide.

## ANNOUNCEMENT

Severe acute respiratory syndrome coronavirus 2 (SARS-CoV-2), a member of the genus *Betacoronavirus*, family *Coronaviridae*, is the causative agent of coronavirus disease 2019 (COVID-19) (1). In Chile, the first confirmed SARS-CoV-2 case was reported on 3 March 2020 (2), and as of 31 January 2022, more than 2.25 million cases had been reported (3).

In the context of the pandemic, genomic surveillance has made it possible to identify circulating variants in certain geographical regions and detect new mutations. It has helped to understand the effect of these changes on the transmission of the virus, the effectiveness of diagnostic and therapeutic methods, and its impact on public health (4). Accordingly, the Chilean Ministry of Health implemented the SARS-CoV-2 epidemiological surveillance project to increase the amount of sequencing data available on SARS-CoV-2 in Chile. Here, we report the genome sequences of 408 SARS-CoV-2 strains from 30 different locations in Araucanía Region, Southern Chile, between August and December 2021. According to epidemiological traits, including hospitalized and nonhospitalized people (5), nasopharyngeal swab samples were collected, and then regional Seremi de Salud personnel selected positive samples by reverse transcription-PCR (RT-PCR) (6), with an average cycle threshold (*C_T_*) value of <30 for genome sequencing. Approval by a research ethics committee for sequencing these strains was not required because these activities were conducted as part of the legislated mandate of the Chilean Ministry of Health (Resolución Exenta no. 403, 21 April 2021).

The RNA extractions for sequencing were performed from respiratory samples by the Zybio, Bioneer, and Smart-32 automated systems using kits recommended by the manufacturers. These RNA extractions were sent to Scientific and Technological Bioresource Nucleus (BIOREN) for whole-genome sequencing. Viral RNA was amplified, and cDNA libraries were prepared using the COVIDseq kit (Illumina) according to the manufacturer’s instructions. Ten sequencing runs were performed using a 600-cycle Illumina 2 × 150-bp kit on an Illumina MiSeq machine, with an average of 41 genomes per run. For data analysis, fastq files were analyzed using the DRAGEN COVID Lineage app version 3.5.3 from the Illumina BaseSpace Sequence Hub using the following settings: Reference genome = NC_045512.2; Enable Trim Sequences = yes; AlignerMinScore = 12; Coverage Threshold = 20; Virus detection Threshold = 5; Enable Duplicate Marking = yes. Finally, the lineage and clade assignments were performed using the Pangolin tool (7), considering the PANGO nomenclature system (8) and viral clades by Nextclade (9). The average genome completeness obtained was 97.19%, with a mean coverage of ∼1,241× and an average GC content of 36.97%.

During the evaluated period, we identified the variants Mu (B.1.621), Lambda (C.37), Gamma (P.1), and Delta (AY.101 AY.102, AY.119, AY.122, AY.20, AY.23, AY.25, AY.26, AY.39, AY.4, AY.43, AY.43.3, AY.46.2, AY.59, AY.7.1, AY.89, B.1.617.2). Of the variant Delta, the lineages B.1.617.2, AY.25 and AY.43 were highlighted as the most prevalent. Table 1 summarizes the information for each sample.

**TABLE 1:** Genomic features of 408 SARS-CoV-2 clinical samples^*a*^

ID	Age (yrs)	Sex	Patient status	Vaccination history	Collection date^*b*^	Lineage	Clade	WHO variant	GC content (%)	Genome completeness (%)	Mean depth of coverage (×)	GISAID ID	GenBank ID	SRA accession
AR-UFRO-0001	47	Female	Alive, not hospitalized	Two doses Sinovac	05-08-21	P.1.15	20J	Gamma	37.77	99.25	1,723	EPI_ISL_4510310	OM146074	SRR18184545
AR-UFRO-0002	32	Male	Alive, not hospitalized	Two doses Pfizer	06-08-21	C.37	21G	Lambda	37.54	98.57	1,417	EPI_ISL_4510311	OM146075	SRR18184544
AR-UFRO-0003	68	Female	Alive, not hospitalized	Two doses Sinovac	05-08-21	P.1.15	20J	Gamma	37.96	99.76	2,100	EPI_ISL_4510312	OM146076	SRR18184505
AR-UFRO-0004	74	Female	Hospitalized	One dose Sinovac	07-08-21	P.1.15	20J	Gamma	37.43	98.31	1,609	EPI_ISL_4510313	OM146077	SRR18184682
AR-UFRO-0005	18	Female	Alive, not hospitalized	Two doses Sinovac	06-08-21	P.1	20J	Not determined	37.3	97.95	1,381	EPI_ISL_4510314	OM146078	SRR18184475
AR-UFRO-0006	30	Female	Alive, not hospitalized	Two doses Sinovac	06-08-21	C.37	21G	Lambda	37.93	99.72	1,564	EPI_ISL_4510315	OM146079	SRR18184600
AR-UFRO-0007	70	Male	Alive, not hospitalized	Not vaccinated	06-08-21	P.1	20J	Gamma	37.93	99.68	2,111	EPI_ISL_4510316	OM146080	SRR18184781
AR-UFRO-0008	79	Female	Alive, not hospitalized	Two doses Sinovac	08-08-21	P.1	20J	Gamma	37.94	99.72	2,070	EPI_ISL_4510317	OM146081	SRR18184770
AR-UFRO-0009	68	Female	Alive, not hospitalized	Two doses Sinovac	07-08-21	P.1	20J	Gamma	37.91	99.52	1,730	EPI_ISL_4510318	OM146082	SRR18184759
AR-UFRO-0010	73	Male	Alive, not hospitalized	Two doses Sinovac	07-08-21	P.1	20J	Gamma	37.66	98.85	1,426	EPI_ISL_4510319	OM146083	SRR18184748
AR-UFRO-0011	18	Male	Alive, not hospitalized	Not vaccinated	06-08-21	B.1.621	21H	Mu	37.96	99.71	1,596	EPI_ISL_4555339	OM146065	SRR18184543
AR-UFRO-0012	42	Female	Alive, not hospitalized	Not vaccinated	06-08-21	P.1.15	20J	Gamma	37.64	98.76	1,666	EPI_ISL_4510320	OM146066	SRR18184532
AR-UFRO-0013	18	Male	Alive, not hospitalized	One dose Sinovac	06-08-21	P.1.15	20J	Gamma	37.4	98.11	1,543	EPI_ISL_4510321	OM146067	SRR18184593
AR-UFRO-0014	51	Male	Alive, not hospitalized	Two doses Sinovac	06-08-21	P.1.15	20J	Gamma	35.12	91.4	235	EPI_ISL_4510322	OM146068	SRR18184582
AR-UFRO-0015	33	Female	Alive, not hospitalized	Two doses Sinovac	07-08-21	P.1.15	20J	Gamma	37.63	98.89	1,816	EPI_ISL_4510323	OM146069	SRR18184571
AR-UFRO-0016	69	Female	Alive, not hospitalized	Two doses Sinovac	08-08-21	P.1	20J	Gamma	37.91	99.54	1,672	EPI_ISL_4510324	OM146070	SRR18184464
AR-UFRO-0017	48	Male	Alive, not hospitalized	Two doses Sinovac	08-08-21	P.1.15	20J	Gamma	37.84	99.38	1,784	EPI_ISL_4510325	OM146071	SRR18184453
AR-UFRO-0018	89	Male	Asymptomatic	Two doses Sinovac	06-08-21	P.1	20J	Gamma	36.57	95.8	1,608	EPI_ISL_4510326	OM146072	SRR18184730
AR-UFRO-0019	75	Male	Asymptomatic	Two doses Sinovac	06-08-21	P.1	20J	Gamma	37.81	99.34	1,955	EPI_ISL_4510327	OM146073	SRR18184719
AR-UFRO-0020	24	Male	Alive, not hospitalized	Two doses Sinovac	21-09-21	P.1	20J	Gamma	37.84	99.48	2,793	EPI_ISL_4957754	OM146084	SRR18184516
AR-UFRO-0021	26	Female	Alive, not hospitalized	Two doses Sinovac	21-09-21	P.1	20J	Gamma	37.93	99.73	2,977	EPI_ISL_4957755	OM146085	SRR18184504
AR-UFRO-0022	29	Male	Alive, not hospitalized	Two doses Pfizer	21-09-21	AY.25.1	21J	Delta	37.7	98.91	2,165	EPI_ISL_4957759	OM146086	SRR18184565
AR-UFRO-0023	35	Male	Alive, not hospitalized	Two doses Sinovac	21-09-21	AY.25.1	21J	Delta	36.88	96.7	1,348	EPI_ISL_4957758	OM146087	SRR18184554
AR-UFRO-0024	43	Male	Alive, not hospitalized	Two doses Sinovac	23-09-21	AY.43	21J	Delta	36.47	95.52	1,397	EPI_ISL_4985032	OM146088	SRR18184447
AR-UFRO-0025	38	Male	Hospitalized	One dose Sinovac	23-09-21	AY.43	21J	Delta	37.03	97.17	1,055	EPI_ISL_4985043	OM146089	SRR18184436
AR-UFRO-0026	38	Female	Alive, not hospitalized	Two doses Sinovac	24-09-21	AY.25.1	21J	Delta	37.26	97.79	1,679	EPI_ISL_4985047	OM146090	SRR18184713
AR-UFRO-0027	37	Male	Alive, not hospitalized	Two doses Pfizer	24-09-21	AY.122	21J	Delta	37.97	99.75	3,051	EPI_ISL_5071343	OM146091	SRR18184702
AR-UFRO-0028	51	Male	Alive, not hospitalized	Two doses Pfizer	24-09-21	AY.122	21J	Delta	37.97	99.72	2,660	EPI_ISL_5071344	OM146160	SRR18184691
AR-UFRO-0029	46	Female	Alive, not hospitalized	Two doses Sinovac	24-09-21	AY.101	21J	Delta	37.35	97.99	1,479	EPI_ISL_4957756	OM146092	SRR18184416
AR-UFRO-0030	49	Male	Alive, not hospitalized	Two doses Sinovac	24-09-21	B.1.621	21H	Mu	37.94	99.72	2,441	EPI_ISL_4985069	OM146093	SRR18184405
AR-UFRO-0031	17	Female	Alive, not hospitalized	Not vaccinated	24-09-21	AY.25.1	21J	Delta	37.56	98.6	2,243	EPI_ISL_4985057	OM146094	SRR18184681
AR-UFRO-0032	62	Female	Hospitalized	Not vaccinated	26-09-21	AY.101	21J	Delta	37.95	99.75	2,779	EPI_ISL_4957757	OM146095	SRR18184670
AR-UFRO-0034	24	Female	Alive, not hospitalized	Two doses Sinovac	26-09-21	AY.25.1	21J	Delta	37.97	99.74	2,475	EPI_ISL_4957751	OM146096	SRR18184635
AR-UFRO-0035	58	Male	Alive, not hospitalized	Two doses Sinovac and one dose Pfizer	28-09-21	AY.25.1	21J	Delta	37.97	99.74	3,229	EPI_ISL_4957760	OM146097	SRR18184624
AR-UFRO-0036	11	Female	Alive, not hospitalized	Not vaccinated	28-09-21	AY.25.1	21J	Delta	35.82	94.09	1,256	EPI_ISL_4957761	OM146098	SRR18184397
AR-UFRO-0037	65	Male	Alive, not hospitalized	Not vaccinated	28-09-21	AY.25.1	21J	Delta	36.22	94.77	1,186	EPI_ISL_4957762	OM146099	SRR18184794
AR-UFRO-0039	58	Female	Alive, not hospitalized	Two doses Pfizer	28-09-21	AY.25.1	21J	Delta	34.96	91.36	9,53	EPI_ISL_4957763	OM146100	SRR18184663
AR-UFRO-0040	35	Female	Alive, not hospitalized	Two doses Pfizer	28-09-21	AY.25.1	21J	Delta	27.87	72.17	214	EPI_ISL_4957764	OM146101	SRR18184652
AR-UFRO-0041	38	Male	Alive, not hospitalized	Not vaccinated	28-09-21	AY.25.1	21J	Delta	37.93	99.61	2,318	EPI_ISL_4957765	OM146102	SRR18184497
AR-UFRO-0048	61	Female	Alive, not hospitalized	Two doses Sinovac	30-09-21	AY.25.1	21J	Delta	37.17	97.45	1,037	EPI_ISL_5071290	OM146103	SRR18184486
AR-UFRO-0050	55	Female	Alive, not hospitalized	Two doses Pfizer	30-09-21	AY.25.1	21J	Delta	36.9	96.64	959	EPI_ISL_5071291	OM146104	SRR18184474
AR-UFRO-0051	25	Male	Alive, not hospitalized	Not vaccinated	30-09-21	AY.25.1	21J	Delta	36.64	96	838	EPI_ISL_5071292	OM146105	SRR18184609
AR-UFRO-0052	50	Female	Alive, not hospitalized	Not vaccinated	30-09-21	AY.25.1	21J	Delta	37.59	98.62	1,536	EPI_ISL_5071293	OM146106	SRR18184608
AR-UFRO-0053	56	Male	Alive, not hospitalized	Two doses Sinovac	30-09-21	AY.25.1	21J	Delta	37.37	98.04	1,267	EPI_ISL_5071294	OM146107	SRR18184607
AR-UFRO-0054	13	Male	Alive, not hospitalized	One dose Sinovac	30-09-21	AY.25.1	21J	Delta	37.43	98.2	1,083	EPI_ISL_5071295	OM146108	SRR18184606
AR-UFRO-0055	43	Female	Alive, not hospitalized	Two doses Sinovac	30-09-21	AY.25.1	21J	Delta	37.16	97.46	1,081	EPI_ISL_5071296	OM146109	SRR18184605
AR-UFRO-0056	12	Female	Alive, not hospitalized	Two doses Sinovac	30-09-21	AY.25.1	21J	Delta	37.03	97.16	1,331	EPI_ISL_5071297	OM146110	SRR18184604
AR-UFRO-0057	10	Male	Alive, not hospitalized	Not vaccinated	30-09-21	AY.25.1	21J	Delta	37.31	97.87	1,399	EPI_ISL_5071298	OM146111	SRR18184603
AR-UFRO-0058	22	Male	Alive, not hospitalized	One dose Sinovac	30-09-21	AY.122	21J	Delta	37	97.09	865	EPI_ISL_5071299	OM146112	SRR18184602
AR-UFRO-0060	2	Male	Alive, not hospitalized	Not vaccinated	30-09-21	AY.25.1	21J	Delta	37.02	97.02	1,146	EPI_ISL_5071300	OM146113	SRR18184601
AR-UFRO-0061	7	Female	Alive, not hospitalized	Not vaccinated	30-09-21	AY.101	21J	Delta	27.48	71.23	117	EPI_ISL_5071301	OM146114	SRR18184599
AR-UFRO-0062	10	Female	Alive, not hospitalized	Not vaccinated	30-09-21	AY.101	21J	Delta	27.97	72.37	209	EPI_ISL_5071302	OM146115	SRR18184598
AR-UFRO-0064	38	Female	Alive, not hospitalized	Two doses Sinovac	30-09-21	AY.25.1	21J	Delta	36.99	96.95	827	EPI_ISL_5071303	OM146116	SRR18184597
AR-UFRO-0065	42	Female	Alive, not hospitalized	Two doses Sinovac	01-10-21	AY.100	21J	Delta	28.41	73.88	280	EPI_ISL_5071304	OM146117	SRR18184596
AR-UFRO-0066	66	Female	Alive, not hospitalized	Two doses Pfizer	01-10-21	AY.101	21J	Delta	36.82	96.47	1,141	EPI_ISL_5071305	OM146118	SRR18184595
AR-UFRO-0067	68	Female	Alive, not hospitalized	Not vaccinated	01-10-21	AY.101	21J	Delta	34	89.41	643	EPI_ISL_5071306	OM146119	SRR18184594
AR-UFRO-0068	62	Male	Alive, not hospitalized	Two doses Pfizer	01-10-21	AY.25.1	21J	Delta	37.17	97.48	1,169	EPI_ISL_5071307	OM146120	SRR18184785
AR-UFRO-0069	26	Male	Alive, not hospitalized	Not vaccinated	01-10-21	AY.101	21J	Delta	30.24	78.68	670	EPI_ISL_5071308	OM146121	SRR18184784
AR-UFRO-0071	73	Female	Alive, not hospitalized	Two doses Sinovac	02-10-21	AY.25.1	21J	Delta	37.53	98.4	1,159	EPI_ISL_5071309	OM146122	SRR18184783
AR-UFRO-0074	28	Female	Alive, not hospitalized	Two doses Pfizer	03-10-21	AY.25.1	21J	Delta	37.37	97.99	1,691	EPI_ISL_5071310	OM146123	SRR18184782
AR-UFRO-0075	39	Male	Alive, not hospitalized	Two doses Pfizer	03-10-21	AY.25.1	21J	Delta	37.23	97.65	1,277	EPI_ISL_5071311	OM146124	SRR18184780
AR-UFRO-0076	31	Male	Alive, not hospitalized	Two doses Sinovac	01-10-21	AY.25.1	21J	Delta	37.39	98.13	1,903	EPI_ISL_5071312	OM146125	SRR18184779
AR-UFRO-0077	62	Male	Alive, not hospitalized	Two doses Sinovac	29-09-21	AY.122	21J	Delta	37.46	98.3	1,589	EPI_ISL_5071313	OM146126	SRR18184778
AR-UFRO-0078	47	Male	Alive, not hospitalized	Two doses Sinovac	27-09-21	AY.101	21J	Delta	37.22	97.64	768	EPI_ISL_5071314	OM146127	SRR18184777
AR-UFRO-0079	14	Male	Alive, not hospitalized	Not vaccinated	28-09-21	AY.25.1	21J	Delta	37.35	98	1,166	EPI_ISL_5071315	OM146128	SRR18184776
AR-UFRO-0080	47	Female	Asymptomatic	Two doses Sinovac	27-09-21	AY.25.1	21J	Delta	37.17	97.46	1,154	EPI_ISL_5071316	OM146129	SRR18184775
AR-UFRO-0081	45	Female	Alive, not hospitalized	Two doses Sinovac	28-09-21	AY.25.1	21J	Delta	36.93	97.01	985	EPI_ISL_5071317	OM146130	SRR18184774
AR-UFRO-0082	33	Female	Alive, not hospitalized	Not vaccinated	29-09-21	AY.101	21J	Delta	36.73	96.31	798	EPI_ISL_5071318	OM146131	SRR18184773
AR-UFRO-0083	21	Male	Alive, not hospitalized	Not vaccinated	29-09-21	AY.25.1	21J	Delta	37.29	97.75	895	EPI_ISL_5071319	OM146132	SRR18184772
AR-UFRO-0084	80	Female	Hospitalized	Two doses Sinovac	29-09-21	B.1.621	21H	Mu	37.58	98.73	1,150	EPI_ISL_5094642	OM146133	SRR18184771
AR-UFRO-0085	14	Female	Alive, not hospitalized	Not vaccinated	28-09-21	AY.101	21J	Delta	31.89	83.24	439	EPI_ISL_5071320	OM146134	SRR18184769
AR-UFRO-0086	47	Female	Alive, not hospitalized	Two doses Sinovac	29-09-21	AY.25.1	21J	Delta	37.41	98.09	1,480	EPI_ISL_5071321	OM146135	SRR18184768
AR-UFRO-0087	31	Female	Alive, not hospitalized	Two doses Pfizer	29-09-21	AY.25.1	21J	Delta	37.67	98.87	1,551	EPI_ISL_5071322	OM146136	SRR18184767
AR-UFRO-0088	27	Male	Alive, not hospitalized	Not vaccinated	30-09-21	AY.25.1	21J	Delta	37.17	97.47	1,288	EPI_ISL_5071323	OM146137	SRR18184766
AR-UFRO-0089	10	Female	Asymptomatic	Not vaccinated	30-09-21	AY.25.1	21J	Delta	37.64	98.78	1,539	EPI_ISL_5071324	OM146138	SRR18184765
AR-UFRO-0090	7	Male	Alive, not hospitalized	Not vaccinated	30-09-21	AY.25.1	21J	Delta	37.39	98.07	1,601	EPI_ISL_5071325	OM146139	SRR18184764
AR-UFRO-0091	12	Male	Alive, not hospitalized	Not vaccinated	28-09-21	AY.25.1	21J	Delta	37.67	98.85	1,427	EPI_ISL_5071326	OM146140	SRR18184763
AR-UFRO-0092	28	Male	Alive, not hospitalized	Not vaccinated	28-09-21	P.1	20J	Gamma	37.84	99.37	1,221	EPI_ISL_5071327	OM146141	SRR18184762
AR-UFRO-0093	47	Female	Alive, not hospitalized	Two doses Sinovac	29-09-21	AY.25.1	21J	Delta	37.1	97.23	1,585	EPI_ISL_5071328	OM146142	SRR18184761
AR-UFRO-0095	27	Female	Alive, not hospitalized	Two doses Sinovac	29-09-21	P.1	20J	Gamma	37.85	99.49	1,226	EPI_ISL_5071329	OM146143	SRR18184760
AR-UFRO-0096	12	Male	Alive, not hospitalized	Not vaccinated	30-09-21	AY.25.1	21J	Delta	37.3	97.92	1,567	EPI_ISL_5071330	OM146144	SRR18184758
AR-UFRO-0097	48	Male	Alive, not hospitalized	Two doses Sinovac	30-09-21	AY.25.1	21J	Delta	37.06	97.1	869	EPI_ISL_5071331	OM146145	SRR18184757
AR-UFRO-0098	57	Female	Alive, not hospitalized	Two doses Sinovac	30-09-21	AY.25.1	21J	Delta	37.66	98.88	1,595	EPI_ISL_5071332	OM146146	SRR18184756
AR-UFRO-0099	11	Female	Alive, not hospitalized	Not vaccinated	30-09-21	AY.25.1	21J	Delta	37.21	97.55	1,539	EPI_ISL_5071333	OM146147	SRR18184755
AR-UFRO-0100	25	Male	Alive, not hospitalized	Two doses Sinovac	29-09-21	AY.25.1	21J	Delta	37.32	97.9	919	EPI_ISL_5071334	OM146148	SRR18184754
AR-UFRO-0101	9	Female	Asymptomatic	Not vaccinated	30-09-21	AY.25.1	21J	Delta	37.18	97.57	1,486	EPI_ISL_5071335	OM146149	SRR18184753
AR-UFRO-0102	22	Female	Alive, not hospitalized	Two doses Sinovac	01-10-21	AY.25.1	21J	Delta	37.19	97.48	897	EPI_ISL_5071336	OM146150	SRR18184752
AR-UFRO-0103	67	Male	Alive, not hospitalized	Two doses Sinovac	28-09-21	AY.25.1	21J	Delta	37.17	97.45	1,283	EPI_ISL_5071337	OM146151	SRR18184751
AR-UFRO-0104	28	Female	Alive, not hospitalized	Two doses Sinovac	01-10-21	AY.43.3	21J	Delta	37.77	99.18	1,696	EPI_ISL_5071338	OM146152	SRR18184750
AR-UFRO-0105	32	Male	Alive, not hospitalized	Two doses Sinovac	01-10-21	B.1.621	21H	Mu	37.94	99.73	1,322	EPI_ISL_5094643	OM146153	SRR18184749
AR-UFRO-0106	35	Female	Alive, not hospitalized	Not vaccinated	30-09-21	B.1.621	21H	Mu	37.68	98.95	1,167	EPI_ISL_5094644	OM146154	SRR18184747
AR-UFRO-0107	59	Female	Alive, not hospitalized	Not vaccinated	02-10-21	AY.25.1	21J	Delta	37.24	97.57	850	EPI_ISL_5071339	OM146155	SRR18184746
AR-UFRO-0108	59	Male	Alive, not hospitalized	Two doses Pfizer	02-10-21	AY.43.3	21J	Delta	37.66	98.87	1,853	EPI_ISL_5071340	OM146156	SRR18184745
AR-UFRO-0109	27	Male	Alive, not hospitalized	Two doses Sinovac	03-10-21	AY.100	21J	Delta	37.64	98.88	1,748	EPI_ISL_5071341	OM146157	SRR18184744
AR-UFRO-0110	29	Female	Alive, not hospitalized	Two doses Sinovac	03-10-21	AY.100	21J	Delta	37.62	98.84	1,524	EPI_ISL_5094645	OM146158	SRR18184743
AR-UFRO-0111	18	Female	Asymptomatic	Two doses Pfizer	03-10-21	AY.25.1	21J	Delta	37.26	97.71	1,173	EPI_ISL_5071342	OM146159	SRR18184742
AR-UFRO-0113	6	Female	Alive, not hospitalized	Not vaccinated	07-10-21	AY.25.1	21J	Delta	36.65	95.74	172	EPI_ISL_5294205	OM146161	SRR18184741
AR-UFRO-0114	9	Female	Alive, not hospitalized	Not vaccinated	08-10-21	AY.25.1	21J	Delta	36.56	95.74	155	EPI_ISL_5294213	OM146162	SRR18184740
AR-UFRO-0115	56	Female	Alive, not hospitalized	Two doses Sinovac	08-10-21	AY.75	21J	Delta	30.38	78.76	47	EPI_ISL_5294221	OM146163	SRR18184739
AR-UFRO-0116	85	Male	Alive, not hospitalized	Two doses Sinovac	08-10-21	AY.25.1	21J	Delta	36.89	96.51	236	EPI_ISL_5294229	OM146164	SRR18184738
AR-UFRO-0117	52	Female	Alive, not hospitalized	Two doses Sinovac	09-10-21	AY.25.1	21J	Delta	33.52	87.45	113	EPI_ISL_5294233	OM146165	SRR18184542
AR-UFRO-0118	38	Male	Alive, not hospitalized	Two doses Sinovac	09-10-21	AY.25.1	21J	Delta	36.59	95.87	172	EPI_ISL_5294241	OM146166	SRR18184541
AR-UFRO-0119	2	Female	Alive, not hospitalized	Not vaccinated	04-10-21	AY.25.1	21J	Delta	37.14	97.31	172	EPI_ISL_5294246	OM146167	SRR18184540
AR-UFRO-0120	25	Female	Alive, not hospitalized	Two doses Sinovac	04-10-21	AY.25.1	21J	Delta	37.14	97.38	309	EPI_ISL_5294251	OM146168	SRR18184539
AR-UFRO-0121	29	Female	Alive, not hospitalized	Two doses Sinovac	04-10-21	AY.25.1	21J	Delta	37.03	97.01	226	EPI_ISL_5294258	OM146169	SRR18184538
AR-UFRO-0122	37	Female	Alive, not hospitalized	Two doses Sinovac	04-10-21	AY.43.3	21J	Delta	37.14	97.31	292	EPI_ISL_5294264	OM146170	SRR18184537
AR-UFRO-0123	32	Female	Alive, not hospitalized	Two doses Sinovac	04-10-21	AY.25.1	21J	Delta	36.88	96.58	217	EPI_ISL_5294272	OM146171	SRR18184536
AR-UFRO-0124	45	Male	Alive, not hospitalized	One dose AstraZeneca and one dose Pfizer	04-10-21	AY.25.1	21J	Delta	36.68	96.07	215	EPI_ISL_5315247	OM146172	SRR18184535
AR-UFRO-0125	10	Female	Alive, not hospitalized	Not vaccinated	04-10-21	AY.25.1	21J	Delta	36.88	96.6	299	EPI_ISL_5294277	OM146173	SRR18184534
AR-UFRO-0126	52	Male	Alive, not hospitalized	Two doses Sinovac	06-10-21	AY.25.1	21J	Delta	36.87	96.58	251	EPI_ISL_5294284	OM146174	SRR18184533
AR-UFRO-0127	31	Female	Alive, not hospitalized	Two doses Sinovac	06-10-21	AY.100	21J	Delta	36.39	95.08	103	EPI_ISL_5294290	OM146175	SRR18184531
AR-UFRO-0128	66	Female	Alive, not hospitalized	Two doses Sinovac	07-10-21	AY.43.3	21J	Delta	37.12	97.33	264	EPI_ISL_5294297	OM146176	SRR18184530
AR-UFRO-0129	73	Male	Alive, not hospitalized	Two doses Sinovac	07-10-21	AY.43	21J	Delta	29.85	77.73	55	EPI_ISL_5294301	OM146177	SRR18184529
AR-UFRO-0130	28	Male	Alive, not hospitalized	Two doses Sinovac	07-10-21	AY.43.3	21J	Delta	37.53	98.42	271	EPI_ISL_5294308	OM146178	SRR18184528
AR-UFRO-0131	30	Female	Alive, not hospitalized	Two doses Pfizer	05-10-21	AY.25.1	21J	Delta	36.53	95.5	203	EPI_ISL_5294318	OM146179	SRR18184527
AR-UFRO-0132	13	Female	Alive, not hospitalized	Not vaccinated	05-10-21	AY.25.1	21J	Delta	37.19	97.53	297	EPI_ISL_5294320	OM146180	SRR18184526
AR-UFRO-0133	69	Female	Alive, not hospitalized	Two doses Sinovac	04-10-21	AY.122	21J	Delta	36.6	95.75	238	EPI_ISL_5294328	OM146181	SRR18184525
AR-UFRO-0135	58	Female	Alive, not hospitalized	Two doses Sinovac	05-10-21	AY.25.1	21J	Delta	37.14	97.35	199	EPI_ISL_5294331	OM146182	SRR18184524
AR-UFRO-0136	66	Male	Alive, not hospitalized	Two doses Sinovac	05-10-21	AY.25.1	21J	Delta	35.26	92.08	150	EPI_ISL_5294337	OM146183	SRR18184523
AR-UFRO-0137	39	Female	Alive, not hospitalized	Two doses Sinovac	06-10-21	AY.25.1	21J	Delta	37.04	97.02	252	EPI_ISL_5294345	OM146184	SRR18184522
AR-UFRO-0138	32	Male	Alive, not hospitalized	Two doses Sinovac	06-10-21	AY.25.1	21J	Delta	37.15	97.38	274	EPI_ISL_5294355	OM146185	SRR18184592
AR-UFRO-0139	9	Male	Alive, not hospitalized	Not vaccinated	07-10-21	B.1.621	21H	Mu	37.33	97.94	282	EPI_ISL_5315248	OM146186	SRR18184591
AR-UFRO-0140	46	Female	Alive, not hospitalized	Two doses Sinovac	07-10-21	AY.25.1	21J	Delta	36.89	96.47	144	EPI_ISL_5294365	OM146187	SRR18184590
AR-UFRO-0141	39	Male	Alive, not hospitalized	Two doses Sinovac	07-10-21	AY.43	21J	Delta	35.99	94.24	155	EPI_ISL_5294374	OM146188	SRR18184589
AR-UFRO-0142	24	Female	Alive, not hospitalized	Two doses Pfizer	08-10-21	AY.43	21J	Delta	37.37	97.98	239	EPI_ISL_5315249	OM146189	SRR18184588
AR-UFRO-0143	81	Female	Alive, not hospitalized	Two doses Sinovac	08-10-21	AY.43	21J	Delta	33.84	88.3	83	EPI_ISL_5294381	OM146190	SRR18184587
AR-UFRO-0144	30	Male	Alive, not hospitalized	Not vaccinated	07-10-21	AY.100	21J	Delta	37.11	97.37	248	EPI_ISL_5294388	OM146191	SRR18184586
AR-UFRO-0145	60	Female	Alive, not hospitalized	Two doses Sinovac	08-10-21	AY.43.3	21J	Delta	37.16	97.48	289	EPI_ISL_5294396	OM146192	SRR18184585
AR-UFRO-0146	70	Male	Alive, not hospitalized	Two doses Sinovac	07-10-21	AY.122	21J	Delta	37.41	98.2	340	EPI_ISL_5294404	OM146193	SRR18184584
AR-UFRO-0148	57	Female	Alive, not hospitalized	Two doses Sinovac	07-10-21	AY.43.3	21J	Delta	36.34	95.14	216	EPI_ISL_5294412	OM146194	SRR18184583
AR-UFRO-0149	28	Female	Alive, not hospitalized	Two doses Pfizer	06-10-21	AY.122	21J	Delta	37.11	97.38	335	EPI_ISL_5294419	OM146195	SRR18184581
AR-UFRO-0150	24	Male	Alive, not hospitalized	Not vaccinated	05-10-21	AY.43	21J	Delta	29.13	74.74	47	EPI_ISL_5294424	OM146196	SRR18184580
AR-UFRO-0151	36	Male	Alive, not hospitalized	Two doses Sinovac	05-10-21	AY.25.1	21J	Delta	37.15	97.4	210	EPI_ISL_5294430	OM146197	SRR18184579
AR-UFRO-0152	74	Male	Alive, not hospitalized	Two doses Sinovac	08-10-21	AY.122	21J	Delta	31.37	81.89	87	EPI_ISL_5294439	OM146198	SRR18184578
AR-UFRO-0153	71	Male	Alive, not hospitalized	Two doses Sinovac	08-10-21	AY.122	21J	Delta	37.41	98.24	331	EPI_ISL_5294445	OM146199	SRR18184577
AR-UFRO-0154	33	Female	Alive, not hospitalized	One dose Sinovac	08-10-21	AY.25.1	21J	Delta	37.61	98.69	296	EPI_ISL_5294453	OM146200	SRR18184576
AR-UFRO-0155	50	Female	Alive, not hospitalized	Two doses Sinovac	07-10-21	AY.25.1	21J	Delta	36.17	94.68	274	EPI_ISL_5294462	OM146201	SRR18184575
AR-UFRO-0156	8	Female	Alive, not hospitalized	Not vaccinated	06-10-21	AY.25.1	21J	Delta	36.98	96.54	320	EPI_ISL_5294471	OM146202	SRR18184574
AR-UFRO-0157	21	Male	Alive, not hospitalized	Two doses Sinovac	12-10-21	AY.25.1	21J	Delta	37.13	97.35	283	EPI_ISL_5294478	OM146203	SRR18184573
AR-UFRO-0162	26	Male	Alive, not hospitalized	Two doses Sinovac	11-10-21	B.1.617.2	21J	Delta	25.34	65.72	35	EPI_ISL_5294484	OM146204	SRR18184572
AR-UFRO-0163	38	Male	Alive, not hospitalized	Two doses Sinovac	12-10-21	AY.25.1	21J	Delta	32.97	85.96	125	EPI_ISL_5294488	OM146205	SRR18184570
AR-UFRO-0164	76	Male	Alive, not hospitalized	Two doses Sinovac	12-10-21	AY.25.1	21J	Delta	34.87	91.56	166	EPI_ISL_5294497	OM146206	SRR18184473
AR-UFRO-0168	73	Female	Alive, not hospitalized	Two doses Sinovac	12-10-21	AY.25.1	21J	Delta	35.96	94.31	404	EPI_ISL_5524302	OM146213	SRR18184472
AR-UFRO-0169	14	Female	Alive, not hospitalized	Not vaccinated	13-10-21	AY.25.1	21J	Delta	37.59	98.67	1,170	EPI_ISL_5524314	OM146242	SRR18184471
AR-UFRO-0172	34	Female	Alive, not hospitalized	Two doses Pfizer	14-10-21	AY.25.1	21J	Delta	36.4	95.45	569	EPI_ISL_5524327	OM146218	SRR18184470
AR-UFRO-0174	57	Female	Alive, not hospitalized	Two doses Sinovac	14-10-21	AY.101	21J	Delta	37.66	98.93	1,038	EPI_ISL_5524328	OM146261	SRR18184469
AR-UFRO-0175	56	Female	Alive, not hospitalized	Two doses Sinovac	14-10-21	AY.25.1	21J	Delta	37.5	98.43	984	EPI_ISL_5524329	OM146237	SRR18184468
AR-UFRO-0176	77	Male	Alive, not hospitalized	Two doses Sinovac	14-10-21	AY.20	21J	Delta	35.46	92.91	329	EPI_ISL_5524330	OM146210	SRR18184467
AR-UFRO-0178	15	Female	Alive, not hospitalized	Two doses Sinovac	16-10-21	AY.25.1	21J	Delta	36.87	96.64	828	EPI_ISL_5524345	OM146221	SRR18184466
AR-UFRO-0179	63	Female	Alive, not hospitalized	Not vaccinated	16-10-21	AY.25.1	21J	Delta	31.82	83.24	90	EPI_ISL_5524346	OM146208	SRR18184465
AR-UFRO-0181	52	Female	Alive, not hospitalized	Two doses Pfizer	17-10-21	AY.25.1	21J	Delta	36.63	96.17	571	EPI_ISL_5524348	OM146219	SRR18184463
AR-UFRO-0182	26	Female	Alive, not hospitalized	Two doses Pfizer	18-10-21	AY.122	21J	Delta	37.76	99.14	1,070	EPI_ISL_5524349	OM146262	SRR18184462
AR-UFRO-0184	11	Male	Alive, not hospitalized	One dose Sinovac	18-10-21	AY.25.1	21J	Delta	36.93	96.82	917	EPI_ISL_5524350	OM146222	SRR18184461
AR-UFRO-0185	57	Female	Alive, not hospitalized	Two doses Pfizer	18-10-21	AY.25.1	21J	Delta	37.48	98.3	1,210	EPI_ISL_5524351	OM146233	SRR18184460
AR-UFRO-0186	81	Female	Alive, not hospitalized	Two doses Sinovac	18-10-21	AY.25.1	21J	Delta	36.16	94.78	772	EPI_ISL_5524352	OM146216	SRR18184459
AR-UFRO-0187	62	Male	Alive, not hospitalized	Two doses Sinovac	18-10-21	AY.25.1	21J	Delta	35.64	93.18	475	EPI_ISL_5524353	OM146212	SRR18184458
AR-UFRO-0188	14	Female	Alive, not hospitalized	Not vaccinated	18-10-21	AY.129	21J	Delta	35.47	93.03	422	EPI_ISL_5524354	OM146211	SRR18184457
AR-UFRO-0189	47	Male	Alive, not hospitalized	One dose Sinovac	18-10-21	AY.20	21J	Delta	37.95	99.74	1,239	EPI_ISL_5524355	OM146271	SRR18184456
AR-UFRO-0190	61	Female	Alive, not hospitalized	Not vaccinated	18-10-21	AY.20	21J	Delta	37.49	98.49	1,067	EPI_ISL_5524356	OM146239	SRR18184455
AR-UFRO-0191	24	Male	Alive, not hospitalized	Two doses Sinovac	19-10-21	AY.25.1	21J	Delta	37.48	98.34	1,183	EPI_ISL_5524357	OM146235	SRR18184454
AR-UFRO-0193	55	Male	Alive, not hospitalized	Two doses Sinovac	19-10-21	AY.25.1	21J	Delta	34.94	91.71	329	EPI_ISL_5524358	OM146209	SRR18184452
AR-UFRO-0194	32	Male	Alive, not hospitalized	One dose AstraZeneca and one dose Pfizer	19-10-21	AY.25.1	21J	Delta	36.07	94.48	431	EPI_ISL_5524359	OM146214	SRR18184451
AR-UFRO-0195	42	Female	Hospitalized	Not vaccinated	28-09-21	AY.25.1	21J	Delta	37.67	98.84	1,064	EPI_ISL_5524299	OM146245	SRR18184450
AR-UFRO-0196	48	Male	Alive, not hospitalized	Two doses Sinovac	12-10-21	AY.43	21J	Delta	37.67	98.84	1,143	EPI_ISL_5866982	OM146246	SRR18184737
AR-UFRO-0197	43	Male	Alive, not hospitalized	Not vaccinated	12-10-21	AY.43.3	21J	Delta	37.64	98.81	1,078	EPI_ISL_5524303	OM146244	SRR18184736
AR-UFRO-0198	24	Male	Alive, not hospitalized	Two doses CanSino	12-10-21	AY.43.3	21J	Delta	37.33	97.96	765	EPI_ISL_5524304	OM146227	SRR18184735
AR-UFRO-0199	46	Female	Alive, not hospitalized	Two doses Sinovac	11-10-21	AY.25.1	21J	Delta	36.35	95.43	650	EPI_ISL_5524300	OM146217	SRR18184734
AR-UFRO-0200	63	Female	Alive, not hospitalized	Two doses Sinovac	12-10-21	AY.43.3	21J	Delta	37.66	98.86	1,184	EPI_ISL_5524305	OM146257	SRR18184733
AR-UFRO-0201	32	Male	Alive, not hospitalized	Two doses Sinovac	12-10-21	AY.25.1	21J	Delta	37.6	98.59	861	EPI_ISL_5524306	OM146240	SRR18184732
AR-UFRO-0202	46	Male	Alive, not hospitalized	Two doses Pfizer	11-10-21	B.1.621	21H	Mu	37.97	99.74	1,168	EPI_ISL_5866981	OM146272	SRR18184731
AR-UFRO-0203	18	Male	Alive, not hospitalized	Two doses CanSino	12-10-21	B.1.621	21H	Mu	37.87	99.56	1,019	EPI_ISL_5866983	OM146268	SRR18184729
AR-UFRO-0204	30	Male	Alive, not hospitalized	Not vaccinated	11-10-21	AY.100	21J	Delta	37.8	99.36	1,359	EPI_ISL_5524301	OM146267	SRR18184728
AR-UFRO-0205	58	Female	Alive, not hospitalized	Two doses Sinovac	13-10-21	AY.43.3	21J	Delta	37.63	98.75	1,123	EPI_ISL_5524315	OM146243	SRR18184727
AR-UFRO-0206	30	Male	Alive, not hospitalized	Two doses Sinovac	13-10-21	AY.25.1	21J	Delta	37.48	98.35	1,115	EPI_ISL_5524316	OM146236	SRR18184726
AR-UFRO-0207	53	Female	Alive, not hospitalized	Two doses Sinovac	14-10-21	AY.122	21J	Delta	37.9	99.65	1,463	EPI_ISL_5524331	OM146269	SRR18184725
AR-UFRO-0208	22	Female	Alive, not hospitalized	Two doses Sinovac	12-10-21	AY.25.1	21J	Delta	37.16	97.14	873	EPI_ISL_5524307	OM146224	SRR18184724
AR-UFRO-0209	23	Male	Alive, not hospitalized	Two doses CanSino	12-10-21	AY.25.1	21J	Delta	36.18	94.48	502	EPI_ISL_5524308	OM146215	SRR18184723
AR-UFRO-0210	44	Female	Alive, not hospitalized	Two doses Sinovac	13-10-21	AY.43.3	21J	Delta	37.66	98.87	1,391	EPI_ISL_5524317	OM146259	SRR18184722
AR-UFRO-0211	42	Male	Alive, not hospitalized	Two doses Sinovac	13-10-21	AY.43.3	21J	Delta	37.65	98.84	1,046	EPI_ISL_5524318	OM146247	SRR18184721
AR-UFRO-0212	20	Female	Alive, not hospitalized	Two doses Sinovac	13-10-21	AY.43.3	21J	Delta	37.65	98.84	1,178	EPI_ISL_5524319	OM146248	SRR18184720
AR-UFRO-0213	44	Female	Alive, not hospitalized	Two doses Sinovac	13-10-21	AY.43.3	21J	Delta	37.45	98.24	898	EPI_ISL_5524320	OM146232	SRR18184718
AR-UFRO-0214	8	Male	Alive, not hospitalized	One dose Sinovac	13-10-21	AY.43.3	21J	Delta	37.36	98.02	937	EPI_ISL_5524321	OM146228	SRR18184717
AR-UFRO-0215	29	Female	Alive, not hospitalized	Two doses Sinovac	12-10-21	AY.43.3	21J	Delta	37.65	98.84	1,327	EPI_ISL_5524309	OM146249	SRR18184716
AR-UFRO-0216	49	Male	Alive, not hospitalized	Two doses Sinovac	12-10-21	AY.25.1	21J	Delta	37.68	98.85	1,137	EPI_ISL_5524310	OM146253	SRR18184715
AR-UFRO-0217	41	Male	Alive, not hospitalized	Two doses Sinovac	13-10-21	AY.43.3	21J	Delta	37.53	98.46	943	EPI_ISL_5524322	OM146238	SRR18184714
AR-UFRO-0218	48	Female	Alive, not hospitalized	Two doses Pfizer	12-10-21	AY.43	21J	Delta	37.43	98.23	994	EPI_ISL_5866984	OM146231	SRR18184521
AR-UFRO-0219	22	Female	Alive, not hospitalized	Two doses Pfizer	12-10-21	AY.43	21J	Delta	37.25	97.65	843	EPI_ISL_5866985	OM146226	SRR18184520
AR-UFRO-0220	31	Female	Alive, not hospitalized	Not vaccinated	12-10-21	AY.122	21J	Delta	37.64	98.85	1,252	EPI_ISL_5524311	OM146254	SRR18184519
AR-UFRO-0221	32	Male	Alive, not hospitalized	Two doses Sinovac	12-10-21	AY.25.1	21J	Delta	37.78	99.14	1,297	EPI_ISL_5524312	OM146263	SRR18184518
AR-UFRO-0222	59	Male	Alive, not hospitalized	Not vaccinated	12-10-21	AY.25.1	21J	Delta	36.92	96.57	794	EPI_ISL_5524313	OM146220	SRR18184517
AR-UFRO-0223	8	Male	Alive, not hospitalized	Not vaccinated	14-10-21	AY.25.1	21J	Delta	37.39	98.03	929	EPI_ISL_5524332	OM146229	SRR18184515
AR-UFRO-0225	7	Male	Alive, not hospitalized	Not vaccinated	14-10-21	AY.25.1	21J	Delta	37.67	98.84	1,047	EPI_ISL_5524334	OM146250	SRR18184514
AR-UFRO-0226	37	Female	Alive, not hospitalized	Two doses Sinovac	14-10-21	AY.43.3	21J	Delta	37.65	98.87	1,310	EPI_ISL_5524335	OM146260	SRR18184513
AR-UFRO-0227	12	Male	Alive, not hospitalized	One dose Sinovac	14-10-21	B.1.621	21H	Mu	37.96	99.72	1,308	EPI_ISL_5866986	OM146270	SRR18184512
AR-UFRO-0228	48	Female	Alive, not hospitalized	Two doses Sinovac	15-10-21	AY.25.1	21J	Delta	37.77	99.16	1,513	EPI_ISL_5524340	OM146264	SRR18184511
AR-UFRO-0229	27	Male	Alive, not hospitalized	Two doses Sinovac	15-10-21	AY.25.1	21J	Delta	37.05	97.12	688	EPI_ISL_5524341	OM146223	SRR18184510
AR-UFRO-0230	12	Female	Alive, not hospitalized	One dose Pfizer	14-10-21	AY.43	21J	Delta	37.15	97.58	618	EPI_ISL_5524336	OM146225	SRR18184509
AR-UFRO-0231	58	Female	Alive, not hospitalized	Two doses Sinovac	14-10-21	AY.122	21J	Delta	37.96	99.74	1,563	EPI_ISL_5524337	OM146273	SRR18184508
AR-UFRO-0232	65	Male	Alive, not hospitalized	Two doses Sinovac	14-10-21	AY.122	21J	Delta	37.81	99.35	1,453	EPI_ISL_5524338	OM146266	SRR18184507
AR-UFRO-0233	18	Female	Alive, not hospitalized	Two doses Sinovac	15-10-21	AY.122	21J	Delta	37.44	98.33	1,612	EPI_ISL_5524342	OM146234	SRR18184506
AR-UFRO-0234	43	Male	Alive, not hospitalized	Not vaccinated	15-10-21	AY.122	21J	Delta	37.52	98.61	1,335	EPI_ISL_5524343	OM146241	SRR18184503
AR-UFRO-0235	58	Male	Alive, not hospitalized	Two doses Sinovac	15-10-21	AY.25.1	21J	Delta	37.65	98.84	1,331	EPI_ISL_5866987	OM146251	SRR18184502
AR-UFRO-0236	51	Female	Hospitalized	Two doses Sinovac	15-10-21	AY.25.1	21J	Delta	37.66	98.85	1,529	EPI_ISL_5524344	OM146255	SRR18184501
AR-UFRO-0237	33	Male	Alive, not hospitalized	Not vaccinated	13-10-21	AY.25.1	21J	Delta	37.67	98.85	1,485	EPI_ISL_5524323	OM146256	SRR18184500
AR-UFRO-0238	75	Male	Alive, not hospitalized	Two doses Sinovac	13-10-21	AY.25.1	21J	Delta	29.19	75.03	869	EPI_ISL_5524324	OM146207	SRR18184499
AR-UFRO-0239	30	Male	Alive, not hospitalized	One dose Sinovac	14-10-21	AY.25.1	21J	Delta	37.38	98.06	1,040	EPI_ISL_5524339	OM146230	SRR18184498
AR-UFRO-0240	2	Male	Alive, not hospitalized	Not vaccinated	13-10-21	AY.43.3	21J	Delta	37.78	99.17	1,457	EPI_ISL_5524325	OM146265	SRR18184569
AR-UFRO-0241	22	Female	Alive, not hospitalized	Two doses Sinovac	13-10-21	AY.43.3	21J	Delta	37.65	98.86	1,551	EPI_ISL_5524326	OM146258	SRR18184568
AR-UFRO-0243	64	Male	Hospitalized	Two doses Sinovac	16-10-21	AY.25.1	21J	Delta	37.66	98.84	1,358	EPI_ISL_5524347	OM146252	SRR18184567
AR-UFRO-0245	20	Male	Alive, not hospitalized	Two doses Sinovac	19-10-21	AY.122	21J	Delta	35.74	93.6	667	EPI_ISL_5881129	OM146274	SRR18184566
AR-UFRO-0247	41	Female	Alive, not hospitalized	Two doses Sinovac	20-10-21	AY.43.3	21J	Delta	37.18	97.51	1,379	EPI_ISL_5881139	OM146275	SRR18184564
AR-UFRO-0249	7	Female	Alive, not hospitalized	One dose Sinovac	21-10-21	AY.25.1	21J	Delta	36.01	94.42	835	EPI_ISL_5881128	OM146276	SRR18184563
AR-UFRO-0250	4	Male	Alive, not hospitalized	Not vaccinated	21-10-21	AY.25.1	21J	Delta	37.4	98.23	1,724	EPI_ISL_5881136	OM146277	SRR18184562
AR-UFRO-0259	55	Female	Alive, not hospitalized	Two doses Sinovac	24-10-21	AY.25.1	21J	Delta	37.9	99.51	1,567	EPI_ISL_5881108	OM146278	SRR18184561
AR-UFRO-0260	63	Male	Alive, not hospitalized	Two doses Sinovac	24-10-21	AY.25.1	21J	Delta	37.81	99.28	1,830	EPI_ISL_5881109	OM146279	SRR18184560
AR-UFRO-0261	29	Male	Alive, not hospitalized	Two doses Sinovac	24-10-21	AY.122	21J	Delta	37.59	98.65	1,594	EPI_ISL_5881131	OM146280	SRR18184559
AR-UFRO-0262	24	Female	Alive, not hospitalized	Two doses Sinovac	23-10-21	AY.25.1	21J	Delta	29.7	77.09	185	EPI_ISL_5881127	OM146281	SRR18184558
AR-UFRO-0263	35	Male	Alive, not hospitalized	Two doses Sinovac	19-10-21	AY.122	21J	Delta	37.65	98.88	1,649	EPI_ISL_5881111	OM146282	SRR18184557
AR-UFRO-0264	36	Female	Alive, not hospitalized	Not vaccinated	20-10-21	AY.122	21J	Delta	37.63	98.85	1,690	EPI_ISL_5881123	OM146283	SRR18184556
AR-UFRO-0265	12	Male	Alive, not hospitalized	Not vaccinated	18-10-21	AY.122	21J	Delta	37.87	99.53	1,826	EPI_ISL_5881112	OM146284	SRR18184555
AR-UFRO-0266	36	Female	Alive, not hospitalized	Two doses Sinovac	18-10-21	AY.122	21J	Delta	37.5	98.4	1,450	EPI_ISL_5881135	OM146285	SRR18184553
AR-UFRO-0267	36	Male	Alive, not hospitalized	Two doses Sinovac	18-10-21	AY.122	21J	Delta	37.65	98.95	1,719	EPI_ISL_5881113	OM146286	SRR18184552
AR-UFRO-0268	48	Male	Alive, not hospitalized	Two doses Pfizer	18-10-21	AY.43	21J	Delta	37.67	98.87	1,745	EPI_ISL_5881114	OM146287	SRR18184551
AR-UFRO-0269	39	Male	Alive, not hospitalized	Two doses Sinovac	18-10-21	AY.122	21J	Delta	37.66	98.87	1,721	EPI_ISL_5881115	OM146288	SRR18184550
AR-UFRO-0270	36	Female	Alive, not hospitalized	Two doses Sinovac	20-10-21	AY.122	21J	Delta	37.54	98.49	1,331	EPI_ISL_5881132	OM146289	SRR18184549
AR-UFRO-0271	21	Male	Alive, not hospitalized	One dose CanSino	20-10-21	AY.101	21J	Delta	37.46	98.36	1,224	EPI_ISL_5881134	OM146290	SRR18184548
AR-UFRO-0272	22	Male	Alive, not hospitalized	One dose Sinovac	20-10-21	AY.43	21J	Delta	36.94	96.84	1,113	EPI_ISL_5924521	OM146291	SRR18184547
AR-UFRO-0273	85	Male	Alive, not hospitalized	Two doses Sinovac	20-10-21	AY.43	21J	Delta	37.32	97.88	1,280	EPI_ISL_5924519	OM146292	SRR18184546
AR-UFRO-0274	53	Female	Alive, not hospitalized	Two doses Sinovac	20-10-21	AY.43	21J	Delta	37.66	98.84	1,398	EPI_ISL_5924518	OM146293	SRR18184449
AR-UFRO-0275	46	Male	Alive, not hospitalized	Two doses Pfizer	20-10-21	AY.122	21J	Delta	37.34	97.97	1,074	EPI_ISL_5881137	OM146294	SRR18184448
AR-UFRO-0276	13	Female	Alive, not hospitalized	Not vaccinated	20-10-21	AY.122	21J	Delta	37.59	98.75	1,594	EPI_ISL_5881124	OM146295	SRR18184446
AR-UFRO-0279	41	Female	Alive, not hospitalized	Two doses Pfizer	19-10-21	AY.25.1	21J	Delta	33.39	86.75	393	EPI_ISL_5881126	OM146296	SRR18184445
AR-UFRO-0285	35	Female	Alive, not hospitalized	Two doses Sinovac	20-10-21	AY.43.3	21J	Delta	36.86	96.98	1,154	EPI_ISL_5881116	OM146297	SRR18184444
AR-UFRO-0286	54	Female	Alive, not hospitalized	Two doses Pfizer	21-10-21	AY.43.3	21J	Delta	37.95	99.74	2,052	EPI_ISL_5881116	OM146298	SRR18184443
AR-UFRO-0287	39	Female	Alive, not hospitalized	Two doses Sinovac	21-10-21	AY.25.1	21J	Delta	37.94	99.7	2,151	EPI_ISL_5881122	OM146299	SRR18184442
AR-UFRO-0288	41	Male	Alive, not hospitalized	Not vaccinated	22-10-21	AY.25.1	21J	Delta	37.83	99.38	1,754	EPI_ISL_5881117	OM146300	SRR18184441
AR-UFRO-0289	28	Female	Alive, not hospitalized	Not vaccinated	21-10-21	AY.43.3	21J	Delta	37.44	98.46	1,544	EPI_ISL_5881133	OM146301	SRR18184440
AR-UFRO-0290	20	Male	Alive, not hospitalized	Two doses Sinovac	21-10-21	AY.43.3	21J	Delta	37.65	98.85	1,624	EPI_ISL_5881118	OM146302	SRR18184439
AR-UFRO-0291	28	Male	Alive, not hospitalized	Two doses Sinovac	22-10-21	AY.43.3	21J	Delta	37.92	99.66	1,788	EPI_ISL_5881118	OM146303	SRR18184438
AR-UFRO-0292	24	Female	Alive, not hospitalized	Two doses Sinovac	22-10-21	AY.43.3	21J	Delta	37.7	99.04	1,541	EPI_ISL_5881125	OM146304	SRR18184437
AR-UFRO-0293	10	Female	Alive, not hospitalized	Not vaccinated	22-10-21	AY.25.1	21J	Delta	37.25	97.72	1,325	EPI_ISL_5881125	OM146305	SRR18184435
AR-UFRO-0296	34	Female	Alive, not hospitalized	Two doses Pfizer	20-10-21	AY.43.3	21J	Delta	33.85	87.07	518	EPI_ISL_5881125	OM146306	SRR18184434
AR-UFRO-0298	43	Female	Alive, not hospitalized	Two doses Sinovac	21-10-21	AY.122	21J	Delta	34.89	91.24	669	EPI_ISL_5881130	OM146307	SRR18184433
AR-UFRO-0299	40	Male	Alive, not hospitalized	Two doses Sinovac	22-10-21	AY.43.3	21J	Delta	37.96	99.72	1,901	EPI_ISL_5881120	OM146308	SRR18184432
AR-UFRO-0300	10	Male	Alive, not hospitalized	One dose Sinovac	22-10-21	AY.25.1	21J	Delta	37.15	97.42	1,360	EPI_ISL_5881141	OM146309	SRR18184431
AR-UFRO-0301	65	Male	Alive, not hospitalized	Two doses Sinovac	22-10-21	AY.43	21J	Delta	37.21	97.61	1,043	EPI_ISL_5924520	OM146310	SRR18184430
AR-UFRO-0305	5	Female	Alive, not hospitalized	Not vaccinated	25-10-21	AY.122	21J	Delta	37.15	97.49	933	EPI_ISL_5881140	OM146311	SRR18184429
AR-UFRO-0306	76	Female	Alive, not hospitalized	Not vaccinated	25-10-21	AY.25.1	21J	Delta	37.88	99.4	1,764	EPI_ISL_5881110	OM146312	SRR18184428
AR-UFRO-0310	70	Male	Hospitalized	Two doses Sinovac	26-10-21	AY.25.1	21J	Delta	36.91	96.69	792	EPI_ISL_6016330	OM146313	SRR18184427
AR-UFRO-0311	23	Female	Alive, not hospitalized	Not vaccinated	27-10-21	AY.25.1	21J	Delta	37.18	97.48	1,223	EPI_ISL_6016334	OM146314	SRR18184426
AR-UFRO-0313	51	Male	Alive, not hospitalized	Not vaccinated	26-10-21	AY.122	21J	Delta	36.42	95.47	697	EPI_ISL_6016168	OM146315	SRR18184712
AR-UFRO-0315	38	Male	Alive, not hospitalized	One dose Pfizer	27-10-21	AY.122	21J	Delta	36.17	94.68	448	EPI_ISL_6016162	OM146316	SRR18184711
AR-UFRO-0316	32	Male	Alive, not hospitalized	Two doses CanSino	28-10-21	AY.122	21J	Delta	32.79	84.8	286	EPI_ISL_6016344	OM146317	SRR18184710
AR-UFRO-0318	40	Female	Alive, not hospitalized	Two doses Sinovac	28-10-21	AY.25.1	21J	Delta	34.97	90.17	523	EPI_ISL_6016349	OM146318	SRR18184709
AR-UFRO-0321	35	Male	Alive, not hospitalized	Not vaccinated	27-10-21	AY.43	21J	Delta	37.27	97.45	916	EPI_ISL_6016174	OM146319	SRR18184708
AR-UFRO-0322	66	Female	Alive, not hospitalized	Not vaccinated	27-10-21	AY.43	21J	Delta	37.25	97.37	1,187	EPI_ISL_6016177	OM146320	SRR18184707
AR-UFRO-0324	34	Male	Alive, not hospitalized	Two doses Sinovac	29-10-21	AY.25.1	21J	Delta	35.65	93.12	595	EPI_ISL_6016355	OM146321	SRR18184706
AR-UFRO-0327	54	Female	Alive, not hospitalized	Two doses Sinovac	29-10-21	AY.25.1	21J	Delta	37.16	97.43	1,140	EPI_ISL_6016364	OM146322	SRR18184705
AR-UFRO-0329	33	Male	Alive, not hospitalized	Two doses Sinovac	29-10-21	AY.122	21J	Delta	36.9	96.63	512	EPI_ISL_6016181	OM146323	SRR18184704
AR-UFRO-0336	61	Female	Alive, not hospitalized	Two doses Sinovac	31-10-21	AY.25.1	21J	Delta	35.06	91.4	334	EPI_ISL_6016370	OM146324	SRR18184703
AR-UFRO-0338	27	Male	Alive, not hospitalized	Two doses Sinovac	31-10-21	AY.122	21J	Delta	36.95	96.9	687	EPI_ISL_6016377	OM146325	SRR18184701
AR-UFRO-0339	50	Male	Alive, not hospitalized	Not vaccinated	01-11-21	AY.25.1	21J	Delta	37.34	97.94	1,377	EPI_ISL_6016385	OM146326	SRR18184700
AR-UFRO-0340	65	Male	Hospitalized	Two doses Sinovac	27-10-21	AY.43	21J	Delta	37.67	98.87	1,470	EPI_ISL_6016187	OM146327	SRR18184699
AR-UFRO-0342	30	Male	Alive, not hospitalized	Two doses Sinovac	26-10-21	AY.119	21J	Delta	37.65	98.84	1,402	EPI_ISL_6016191	OM146328	SRR18184698
AR-UFRO-0343	44	Male	Hospitalized	Not vaccinated	25-10-21	AY.101	21J	Delta	37.66	98.87	1,257	EPI_ISL_6016124	OM146329	SRR18184697
AR-UFRO-0344	30	Female	Alive, not hospitalized	Two doses Sinovac	25-10-21	AY.43	21J	Delta	37.63	98.29	1,429	EPI_ISL_6016137	OM146330	SRR18184696
AR-UFRO-0345	61	Female	Hospitalized	Not vaccinated	25-10-21	AY.25.1	21J	Delta	37.54	98.49	1,289	EPI_ISL_6016196	OM146331	SRR18184695
AR-UFRO-0350	37	Male	Alive, not hospitalized	Not vaccinated	26-10-21	AY.102	21J	Delta	37.59	98.74	1,477	EPI_ISL_6016145	OM146332	SRR18184694
AR-UFRO-0351	31	Female	Alive, not hospitalized	Two doses Sinovac	26-10-21	AY.43.3	21J	Delta	37.03	97.09	770	EPI_ISL_6016151	OM146333	SRR18184693
AR-UFRO-0352	34	Male	Alive, not hospitalized	Two doses Sinovac	26-10-21	AY.43.3	21J	Delta	37.27	97.72	1,061	EPI_ISL_6016317	OM146334	SRR18184692
AR-UFRO-0353	32	Female	Hospitalized	Two doses Sinovac	27-10-21	AY.25.1	21J	Delta	37.34	97.91	1,311	EPI_ISL_6016202	OM146335	SRR18184690
AR-UFRO-0354	39	Male	Alive, not hospitalized	Two doses Sinovac	26-10-21	AY.25.1	21J	Delta	37.59	98.68	1,300	EPI_ISL_6016321	OM146336	SRR18184425
AR-UFRO-0355	54	Male	Alive, not hospitalized	Two doses Sinovac	26-10-21	AY.25.1	21J	Delta	37.67	98.87	1,559	EPI_ISL_6016324	OM146337	SRR18184424
AR-UFRO-0356	35	Male	Alive, not hospitalized	Two doses Pfizer	28-10-21	AY.25.1	21J	Delta	37.53	98.43	1,547	EPI_ISL_6016309	OM146338	SRR18184423
AR-UFRO-0357	32	Female	Hospitalized	Two doses Sinovac	28-10-21	AY.25.1	21J	Delta	37.84	99.37	1,589	EPI_ISL_6016209	OM146339	SRR18184422
AR-UFRO-0362	41	Male	Alive, not hospitalized	Two doses Sinovac	26-10-21	AY.25.1	21J	Delta	36.93	96.71	893	EPI_ISL_6016307	OM146340	SRR18184421
AR-UFRO-0364	28	Male	Alive, not hospitalized	Two doses Sinovac	26-10-21	AY.122	21J	Delta	37.49	98.37	1,390	EPI_ISL_6016218	OM146341	SRR18184420
AR-UFRO-0365	45	Male	Alive, not hospitalized	Two doses Sinovac	27-10-21	AY.122	21J	Delta	37.41	98.18	1,327	EPI_ISL_6016222	OM146342	SRR18184419
AR-UFRO-0366	13	Female	Alive, not hospitalized	Two doses Sinovac	27-10-21	AY.43.3	21J	Delta	37.19	97.58	1,241	EPI_ISL_6016230	OM146343	SRR18184418
AR-UFRO-0367	14	Female	Alive, not hospitalized	Two doses Pfizer	29-10-21	AY.43.3	21J	Delta	37.25	97.68	1,171	EPI_ISL_6016237	OM146344	SRR18184417
AR-UFRO-0368	47	Female	Alive, not hospitalized	Two doses Pfizer	28-10-21	AY.43.3	21J	Delta	37.15	97.48	938	EPI_ISL_6016244	OM146345	SRR18184415
AR-UFRO-0369	40	Female	Alive, not hospitalized	Two doses Sinovac	28-10-21	AY.43.3	21J	Delta	37.66	98.87	1,413	EPI_ISL_6016251	OM146346	SRR18184414
AR-UFRO-0370	27	Male	Alive, not hospitalized	Not vaccinated	28-10-21	AY.122	21J	Delta	37.65	98.87	1,440	EPI_ISL_6016259	OM146347	SRR18184413
AR-UFRO-0371	40	Female	Alive, not hospitalized	Not vaccinated	28-10-21	AY.43.3	21J	Delta	37.75	99.16	1,612	EPI_ISL_6016266	OM146348	SRR18184412
AR-UFRO-0372	20	Male	Alive, not hospitalized	One dose Pfizer	28-10-21	AY.122	21J	Delta	37.44	98.27	1,182	EPI_ISL_6016272	OM146349	SRR18184411
AR-UFRO-0373	37	Female	Alive, not hospitalized	Two doses Sinovac	28-10-21	AY.43.3	21J	Delta	37.66	98.87	1,492	EPI_ISL_6016277	OM146350	SRR18184410
AR-UFRO-0374	23	Female	Alive, not hospitalized	Two doses Pfizer	27-10-21	AY.119	21J	Delta	37.41	98.13	1,122	EPI_ISL_6016284	OM146351	SRR18184409
AR-UFRO-0375	34	Male	Alive, not hospitalized	Two doses Sinovac	27-10-21	AY.25.1	21J	Delta	37.66	98.87	1,452	EPI_ISL_6016288	OM146352	SRR18184408
AR-UFRO-0376	51	Female	Alive, not hospitalized	Two doses Pfizer	27-10-21	AY.119	21J	Delta	37.65	98.85	1,421	EPI_ISL_6016295	OM146353	SRR18184407
AR-UFRO-0377	48	Male	Alive, not hospitalized	Not vaccinated	27-10-21	AY.43.3	21J	Delta	36.9	96.75	939	EPI_ISL_6016299	OM146354	SRR18184406
AR-UFRO-0378	13	Female	Alive, not hospitalized	Not vaccinated	27-10-21	AY.43.3	21J	Delta	36.97	96.98	873	EPI_ISL_6016160	OM146355	SRR18184404
AR-UFRO-0380	27	Male	Alive, not hospitalized	Two doses CanSino	02-11-21	AY.25.1	21J	Delta	36.22	95.11	731	EPI_ISL_6309830	OM146356	SRR18184403
AR-UFRO-0381	50	Male	Alive, not hospitalized	Two doses Sinovac	02-11-21	AY.43.3	21J	Delta	37.96	99.73	1,377	EPI_ISL_6309831	OM146357	SRR18184402
AR-UFRO-0382	31	Female	Alive, not hospitalized	One dose Sinovac	04-10-21	AY.122	21J	Delta	32.91	86.46	207	EPI_ISL_6309832	OM146358	SRR18184689
AR-UFRO-0383	51	Male	Hospitalized	Two doses Sinovac	07-10-21	AY.25.1	21J	Delta	33.56	88.08	153	EPI_ISL_6309833	OM146359	SRR18184688
AR-UFRO-0384	68	Female	Alive, not hospitalized	Two doses Sinovac	04-11-21	AY.102	21J	Delta	36.55	95.98	774	EPI_ISL_6309834	OM146360	SRR18184687
AR-UFRO-0385	28	Male	Alive, not hospitalized	Two doses Pfizer	04-11-21	AY.25.1	21J	Delta	36.84	96.5	979	EPI_ISL_6309835	OM146361	SRR18184686
AR-UFRO-0386	42	Male	Alive, not hospitalized	Two doses Sinovac	04-11-21	AY.25.1	21J	Delta	37.97	99.71	1,612	EPI_ISL_6309836	OM146362	SRR18184685
AR-UFRO-0387	45	Male	Alive, not hospitalized	Two doses Sinovac	04-11-21	AY.25.1	21J	Delta	37.68	98.87	932	EPI_ISL_6309837	OM146363	SRR18184684
AR-UFRO-0388	31	Female	Alive, not hospitalized	Two doses Pfizer	06-11-21	AY.25.1	21J	Delta	37.97	99.74	1,795	EPI_ISL_6309838	OM146364	SRR18184683
AR-UFRO-0389	39	Male	Alive, not hospitalized	Two doses Sinovac	05-11-21	AY.43	21J	Delta	37.65	98.85	1,341	EPI_ISL_6309839	OM146365	SRR18184680
AR-UFRO-0390	33	Female	Alive, not hospitalized	Not vaccinated	05-11-21	AY.43.3	21J	Delta	37.74	99.14	1,627	EPI_ISL_6309840	OM146366	SRR18184679
AR-UFRO-0391	16	Male	Alive, not hospitalized	Not vaccinated	05-11-21	AY.43.3	21J	Delta	37.69	98.99	1,239	EPI_ISL_6309841	OM146367	SRR18184678
AR-UFRO-0392	7	Female	Alive, not hospitalized	Not vaccinated	05-11-21	AY.43.3	21J	Delta	37.95	99.73	1,677	EPI_ISL_6309842	OM146368	SRR18184677
AR-UFRO-0393	36	Female	Alive, not hospitalized	Two doses Pfizer	05-11-21	AY.101	21J	Delta	31.4	81.66	111	EPI_ISL_6309843	OM146369	SRR18184676
AR-UFRO-0394	42	Female	Alive, not hospitalized	Two doses Pfizer	05-11-21	AY.25.1	21J	Delta	37.98	99.75	1,996	EPI_ISL_6309844	OM146370	SRR18184675
AR-UFRO-0395	54	Male	Alive, not hospitalized	Two doses Pfizer	06-11-21	AY.43.3	21J	Delta	37.65	98.86	1,261	EPI_ISL_6309845	OM146371	SRR18184674
AR-UFRO-0396	88	Female	Hospitalized	Two doses Sinovac	06-11-21	AY.25.1	21J	Delta	37.97	99.74	2,094	EPI_ISL_6309846	OM146372	SRR18184673
AR-UFRO-0397	26	Male	Alive, not hospitalized	Two doses Sinovac	06-11-21	AY.43.3	21J	Delta	37.95	99.72	1,885	EPI_ISL_6309847	OM146373	SRR18184672
AR-UFRO-0398	37	Male	Alive, not hospitalized	Two doses Sinovac	06-11-21	AY.43.3	21J	Delta	37.81	99.32	1,513	EPI_ISL_6309848	OM146374	SRR18184671
AR-UFRO-0399	24	Male	Alive, not hospitalized	Not vaccinated	06-11-21	AY.43.3	21J	Delta	37.96	99.73	1,788	EPI_ISL_6309849	OM146375	SRR18184669
AR-UFRO-0400	6	Male	Alive, not hospitalized	Not vaccinated	06-11-21	AY.101	21J	Delta	37.9	99.58	1,718	EPI_ISL_6309850	OM146376	SRR18184668
AR-UFRO-0401	37	Female	Alive, not hospitalized	Two doses Sinovac	06-11-21	AY.100	21J	Delta	37.95	99.74	1,988	EPI_ISL_6309851	OM146377	SRR18184667
AR-UFRO-0402	22	Male	Alive, not hospitalized	Two doses Sinovac	06-11-21	AY.43.3	21J	Delta	37.87	99.47	1,495	EPI_ISL_6309852	OM146378	SRR18184666
AR-UFRO-0403	44	Male	Alive, not hospitalized	Two doses Sinovac	06-11-21	AY.100	21J	Delta	37.89	99.51	1,449	EPI_ISL_6309853	OM146379	SRR18184641
AR-UFRO-0404	48	Male	Alive, not hospitalized	Two doses Sinovac	06-11-21	AY.43.3	21J	Delta	37.96	99.73	1,899	EPI_ISL_6309854	OM146380	SRR18184640
AR-UFRO-0405	28	Female	Alive, not hospitalized	Not vaccinated	06-11-21	AY.102	21J	Delta	37.96	99.73	1,866	EPI_ISL_6309855	OM146381	SRR18184639
AR-UFRO-0406	58	Female	Alive, not hospitalized	Two doses Sinovac	06-11-21	AY.25.1	21J	Delta	37.66	98.86	1,368	EPI_ISL_6309856	OM146382	SRR18184638
AR-UFRO-0407	63	Female	Alive, not hospitalized	Two doses Sinovac	05-11-21	AY.25.1	21J	Delta	37.67	98.84	1,143	EPI_ISL_6309857	OM146383	SRR18184637
AR-UFRO-0408	41	Male	Alive, not hospitalized	Two doses Sinovac	06-11-21	AY.43	21J	Delta	37.66	98.87	1,460	EPI_ISL_6309858	OM146384	SRR18184636
AR-UFRO-0409	35	Male	Alive, not hospitalized	Two doses CanSino	05-11-21	AY.43.3	21J	Delta	37.64	98.84	1,225	EPI_ISL_6309859	OM146385	SRR18184634
AR-UFRO-0410	25	Female	Alive, not hospitalized	Two doses Sinovac	05-11-21	AY.43.3	21J	Delta	37.96	99.74	1,553	EPI_ISL_6309860	OM146386	SRR18184633
AR-UFRO-0411	86	Male	Alive, not hospitalized	Not vaccinated	05-11-21	AY.102	21J	Delta	37.96	99.73	1,547	EPI_ISL_6309861	OM146387	SRR18184632
AR-UFRO-0412	55	Male	Alive, not hospitalized	Two doses Sinovac	05-11-21	AY.122	21J	Delta	37.64	98.87	1,222	EPI_ISL_6309862	OM146388	SRR18184631
AR-UFRO-0413	9	Female	Alive, not hospitalized	One dose Sinovac	06-11-21	AY.43.3	21J	Delta	36.19	95.02	838	EPI_ISL_6309863	OM146389	SRR18184630
AR-UFRO-0414	5	Male	Alive, not hospitalized	Not vaccinated	07-11-21	AY.102	21J	Delta	37.94	99.68	1,725	EPI_ISL_6309864	OM146390	SRR18184629
AR-UFRO-0415	63	Male	Alive, not hospitalized	Two doses Pfizer	07-11-21	AY.43.3	21J	Delta	37.96	99.74	1,559	EPI_ISL_6309865	OM146391	SRR18184628
AR-UFRO-0416	53	Male	Alive, not hospitalized	Two doses Sinovac	07-11-21	AY.43.3	21J	Delta	37.96	99.73	1,684	EPI_ISL_6309866	OM146392	SRR18184627
AR-UFRO-0417	22	Female	Alive, not hospitalized	Two doses Sinovac	07-11-21	AY.43.3	21J	Delta	37.95	99.71	1,542	EPI_ISL_6309867	OM146393	SRR18184626
AR-UFRO-0418	28	Male	Alive, not hospitalized	Not vaccinated	07-11-21	AY.43.3	21J	Delta	37.95	99.69	1,349	EPI_ISL_6309868	OM146394	SRR18184625
AR-UFRO-0419	37	Male	Alive, not hospitalized	Two doses Sinovac	07-11-21	AY.43	21J	Delta	37.94	99.65	1,190	EPI_ISL_6309869	OM146395	SRR18184623
AR-UFRO-0420	31	Female	Alive, not hospitalized	Two doses Sinovac	07-11-21	AY.43.3	21J	Delta	37.96	99.74	1,556	EPI_ISL_6309870	OM146396	SRR18184622
AR-UFRO-0421	27	Female	Alive, not hospitalized	One dose Pfizer	06-11-21	AY.101	21J	Delta	37.96	99.71	1,466	EPI_ISL_6309871	OM146397	SRR18184621
AR-UFRO-0422	69	Male	Hospitalized	Two doses Sinovac	06-11-21	AY.101	21J	Delta	37.96	99.74	1,728	EPI_ISL_6309872	OM146398	SRR18184620
AR-UFRO-0423	22	Female	Alive, not hospitalized	Two doses CanSino	09-11-21	AY.119.1	21J	Delta	37.64	98.94	1,389	EPI_ISL_6605781	OM146399	SRR18184619
AR-UFRO-0424	77	Male	Alive, not hospitalized	Two doses Sinovac	09-11-21	AY.43.3	21J	Delta	37.65	98.85	1,571	EPI_ISL_6605787	OM146400	SRR18184618
AR-UFRO-0425	23	Male	Hospitalized	Two doses Sinovac	11-11-21	AY.43.3	21J	Delta	37.25	97.67	767	EPI_ISL_6605794	OM146401	SRR18184401
AR-UFRO-0426	25	Female	Alive, not hospitalized	One dose Pfizer	09-11-21	AY.43.3	21J	Delta	37.36	98.06	1,180	EPI_ISL_6605802	OM146402	SRR18184400
AR-UFRO-0427	60	Male	Alive, not hospitalized	Two doses Sinovac	10-11-21	AY.25.1	21J	Delta	37.53	98.39	1,347	EPI_ISL_6605812	OM146403	SRR18184399
AR-UFRO-0428	53	Male	Alive, not hospitalized	Two doses Pfizer	09-11-21	AY.25.1	21J	Delta	37.74	99.04	1,498	EPI_ISL_6605816	OM146404	SRR18184398
AR-UFRO-0429	51	Female	Alive, not hospitalized	Two doses Sinovac	10-11-21	AY.43.3	21J	Delta	37.65	98.84	1,736	EPI_ISL_6605825	OM146405	SRR18184396
AR-UFRO-0430	45	Male	Alive, not hospitalized	Two doses Pfizer	10-11-21	AY.102	21J	Delta	37.64	98.84	1744	EPI_ISL_6605832	OM146406	SRR18184395
AR-UFRO-0431	53	Female	Alive, not hospitalized	Two doses Sinovac	11-11-21	AY.43.3	21J	Delta	37.66	98.84	1,410	EPI_ISL_6605840	OM146407	SRR18184394
AR-UFRO-0432	36	Male	Alive, not hospitalized	Not vaccinated	09-11-21	AY.43	21J	Delta	37.47	98.04	996	EPI_ISL_6605846	OM146408	SRR18184393
AR-UFRO-0433	38	Female	Alive, not hospitalized	Not vaccinated	09-11-21	AY.43	21J	Delta	37.68	98.65	1,581	EPI_ISL_6605853	OM146409	SRR18184392
AR-UFRO-0434	63	Female	Alive, not hospitalized	Two doses Sinovac	10-11-21	AY.43	21J	Delta	37.95	99.7	1,530	EPI_ISL_6722292	OM146410	SRR18184391
AR-UFRO-0435	18	Female	Alive, not hospitalized	Two doses Pfizer	09-11-21	AY.25.1	21J	Delta	37.67	98.84	1,503	EPI_ISL_6605861	OM146411	SRR18184390
AR-UFRO-0436	27	Female	Alive, not hospitalized	Two doses Sinovac	08-11-21	AY.43.3	21J	Delta	37.65	98.84	1,599	EPI_ISL_6605869	OM146412	SRR18184389
AR-UFRO-0437	34	Male	Alive, not hospitalized	Two doses Sinovac	09-11-21	AY.43.3	21J	Delta	37.65	98.84	1,380	EPI_ISL_6605879	OM146413	SRR18184388
AR-UFRO-0438	24	Male	Hospitalized	Not vaccinated	12-11-21	AY.43.3	21J	Delta	37.12	97.3	935	EPI_ISL_6605886	OM146414	SRR18184387
AR-UFRO-0439	56	Male	Alive, not hospitalized	Two doses Sinovac	10-11-21	AY.101	21J	Delta	37.66	98.84	1,581	EPI_ISL_6605895	OM146415	SRR18184793
AR-UFRO-0440	50	Female	Alive, not hospitalized	Two doses Pfizer	10-11-21	AY.43.3	21J	Delta	37.96	99.74	1,706	EPI_ISL_6605903	OM146416	SRR18184792
AR-UFRO-0441	39	Male	Alive, not hospitalized	Two doses Sinovac	11-11-21	AY.43	21J	Delta	37.65	98.89	1,371	EPI_ISL_6605910	OM146417	SRR18184791
AR-UFRO-0442	23	Female	Alive, not hospitalized	Two doses Pfizer	11-11-21	AY.43.3	21J	Delta	37.94	99.68	1,695	EPI_ISL_6605915	OM146418	SRR18184790
AR-UFRO-0443	22	Female	Alive, not hospitalized	One dose Sinovac	11-11-21	AY.43.3	21J	Delta	37.18	97.63	1,013	EPI_ISL_6605925	OM146419	SRR18184789
AR-UFRO-0444	19	Female	Alive, not hospitalized	Two doses Pfizer	12-11-21	AY.43.3	21J	Delta	37.65	98.88	1,311	EPI_ISL_6605934	OM146420	SRR18184788
AR-UFRO-0445	24	Male	Alive, not hospitalized	Two doses Pfizer	12-11-21	AY.43.3	21J	Delta	37.34	97.95	1,081	EPI_ISL_6605940	OM146421	SRR18184787
AR-UFRO-0446	54	Female	Alive, not hospitalized	Not vaccinated	12-11-21	AY.119.1	21J	Delta	37.92	99.59	1,493	EPI_ISL_6605951	OM146422	SRR18184786
AR-UFRO-0447	36	Male	Alive, not hospitalized	Two doses Pfizer	13-11-21	AY.25.1	21J	Delta	37.39	98.06	1,122	EPI_ISL_6605955	OM146423	SRR18184665
AR-UFRO-0448	7	Female	Alive, not hospitalized	One dose Sinovac	12-11-21	AY.25.1	21J	Delta	37.45	98.2	1,146	EPI_ISL_6605963	OM146424	SRR18184664
AR-UFRO-0449	23	Female	Alive, not hospitalized	Two doses Sinovac	13-11-21	AY.43.3	21J	Delta	37.6	98.79	1,237	EPI_ISL_6605971	OM146425	SRR18184662
AR-UFRO-0450	12	Male	Alive, not hospitalized	One dose Sinovac	13-11-21	AY.101	21J	Delta	37.83	99.46	1,543	EPI_ISL_6605977	OM146426	SRR18184661
AR-UFRO-0451	25	Female	Alive, not hospitalized	Two doses Sinovac	13-11-21	AY.102	21J	Delta	37.65	98.88	1,320	EPI_ISL_6605986	OM146427	SRR18184660
AR-UFRO-0452	41	Female	Alive, not hospitalized	Two doses Sinovac	13-11-21	AY.25.1	21J	Delta	37.66	98.79	1,208	EPI_ISL_6605990	OM146428	SRR18184659
AR-UFRO-0453	48	Female	Alive, not hospitalized	Two doses Sinovac	12-11-21	AY.43.3	21J	Delta	37.34	97.97	1,013	EPI_ISL_6605999	OM146429	SRR18184658
AR-UFRO-0454	48	Female	Alive, not hospitalized	Two doses Sinovac	14-11-21	AY.101	21J	Delta	37.95	99.7	1,583	EPI_ISL_6606011	OM146430	SRR18184657
AR-UFRO-0455	29	Male	Alive, not hospitalized	Two doses Sinovac	14-11-21	AY.102	21J	Delta	37.65	98.83	1,189	EPI_ISL_6606018	OM146431	SRR18184656
AR-UFRO-0456	20	Male	Hospitalized	Not vaccinated	14-11-21	AY.43.3	21J	Delta	37.65	98.93	1,572	EPI_ISL_6606025	OM146432	SRR18184655
AR-UFRO-0457	52	Female	Hospitalized	Not vaccinated	14-11-21	AY.43.3	21J	Delta	37.66	98.91	1,484	EPI_ISL_6606031	OM146433	SRR18184654
AR-UFRO-0459	8	Male	Alive, not hospitalized	Not vaccinated	12-11-21	AY.25.1	21J	Delta	37.48	98.39	1,337	EPI_ISL_6606041	OM146434	SRR18184653
AR-UFRO-0460	31	Male	Alive, not hospitalized	Two doses Sinovac	18-11-21	AY.101	21J	Delta	37.96	99.71	1,443	EPI_ISL_7698030	OM146435	SRR18184651
AR-UFRO-0461	32	Female	Hospitalized	Two doses Sinovac	21-11-21	AY.25.1	21J	Delta	37.9	99.53	1,363	EPI_ISL_7698032	OM146436	SRR18184650
AR-UFRO-0462	86	Female	Hospitalized	Two doses Sinovac	19-11-21	AY.43.3	21J	Delta	37.87	99.44	1,272	EPI_ISL_7698033	OM146437	SRR18184649
AR-UFRO-0463	60	Male	Hospitalized	Two doses AstraZeneca	17-11-21	AY.102	21J	Delta	36.99	97.37	537	EPI_ISL_7698034	OM146438	SRR18184648
AR-UFRO-0464	25	Female	Hospitalized	Not vaccinated	17-11-21	AY.25.1	21J	Delta	37.67	99.07	843	EPI_ISL_7698038	OM146439	SRR18184647
AR-UFRO-0465	24	Male	Alive, not hospitalized	Two doses Sinovac	19-11-21	AY.25.1	21J	Delta	37.68	98.92	1,388	EPI_ISL_7698040	OM146440	SRR18184646
AR-UFRO-0466	53	Male	Alive, not hospitalized	Two doses Sinovac	19-11-21	AY.122	21J	Delta	37.94	99.81	1,690	EPI_ISL_7698043	OM146441	SRR18184645
AR-UFRO-0467	30	Male	Alive, not hospitalized	Two doses Sinovac	18-11-21	AY.122	21J	Delta	37.34	98.06	690	EPI_ISL_7698045	OM146442	SRR18184644
AR-UFRO-0468	56	Female	Hospitalized	Two doses Sinovac	22-11-21	AY.122	21J	Delta	37.94	99.71	1,288	EPI_ISL_7698048	OM146443	SRR18184643
AR-UFRO-0469	23	Male	Alive, not hospitalized	Two doses Sinovac	21-11-21	AY.43.3	21J	Delta	37.88	99.51	1,217	EPI_ISL_7698050	OM146444	SRR18184642
AR-UFRO-0470	39	Female	Alive, not hospitalized	One dose Sinovac	19-11-21	AY.43	21J	Delta	35.63	93.53	456	EPI_ISL_7698053	OM146445	SRR18184496
AR-UFRO-0473	27	Female	Alive, not hospitalized	Two doses Sinovac	22-11-21	AY.43.3	21J	Delta	37.6	98.69	842	EPI_ISL_7698055	OM146472	SRR18184495
AR-UFRO-0474	49	Male	Alive, not hospitalized	Two doses Sinovac	28-11-21	AY.25.1	21J	Delta	35.14	92.26	487	EPI_ISL_7698057	OM146446	SRR18184494
AR-UFRO-0477	29	Male	Alive, not hospitalized	Two doses Sinovac	27-11-21	AY.43.3	21J	Delta	37.87	99.46	1,025	EPI_ISL_7698060	OM146447	SRR18184493
AR-UFRO-0478	54	Female	Alive, not hospitalized	Not vaccinated	26-11-21	AY.122	21J	Delta	37.82	99.27	1,194	EPI_ISL_7698062	OM146448	SRR18184492
AR-UFRO-0479	21	Female	Alive, not hospitalized	Two doses Pfizer	26-11-21	AY.43.3	21J	Delta	37.96	99.82	1,540	EPI_ISL_7698065	OM146449	SRR18184491
AR-UFRO-0480	71	Female	Alive, not hospitalized	Two doses Sinovac	26-11-21	AY.43.3	21J	Delta	37.65	98.85	1,065	EPI_ISL_7698067	OM146450	SRR18184490
AR-UFRO-0481	18	Male	Alive, not hospitalized	Two doses Sinovac	26-11-21	AY.43.3	21J	Delta	37.84	99.52	1,027	EPI_ISL_7698070	OM146451	SRR18184489
AR-UFRO-0482	63	Male	Alive, not hospitalized	Two doses Sinovac	27-11-21	AY.122	21J	Delta	37.94	99.8	1,407	EPI_ISL_7698073	OM146452	SRR18184488
AR-UFRO-0483	75	Female	Alive, not hospitalized	Two doses Sinovac	26-11-21	AY.43	21J	Delta	37.79	98.93	883	EPI_ISL_7698075	OM146453	SRR18184487
AR-UFRO-0484	28	Male	Alive, not hospitalized	Two doses Sinovac	28-11-21	AY.122	21J	Delta	37.95	99.73	1,502	EPI_ISL_7698077	OM146454	SRR18184485
AR-UFRO-0485	49	Male	Alive, not hospitalized	Two doses Sinovac	29-11-21	AY.102	21J	Delta	37.95	99.83	1,718	EPI_ISL_7698080	OM146455	SRR18184484
AR-UFRO-0486	10	Female	Alive, not hospitalized	One dose Sinovac	29-11-21	AY.122	21J	Delta	37.96	99.82	1,427	EPI_ISL_7698082	OM146456	SRR18184483
AR-UFRO-0487	26	Female	Alive, not hospitalized	Two doses CanSino	29-11-21	AY.43.3	21J	Delta	37.9	99.58	1,372	EPI_ISL_7698085	OM146457	SRR18184482
AR-UFRO-0488	26	Male	Alive, not hospitalized	Two doses Sinovac	01-12-21	AY.43.3	21J	Delta	37.67	98.81	937	EPI_ISL_7698087	OM146458	SRR18184481
AR-UFRO-0489	30	Male	Alive, not hospitalized	Two doses CanSino	04-12-21	AY.43.3	21J	Delta	37.81	99.25	1,140	EPI_ISL_7698089	OM146459	SRR18184480
AR-UFRO-0490	25	Female	Alive, not hospitalized	Two doses Pfizer	04-12-21	AY.122	21J	Delta	37.94	99.68	1,409	EPI_ISL_7698092	OM146460	SRR18184479
AR-UFRO-0491	68	Male	Alive, not hospitalized	Two doses Sinovac	04-12-21	AY.43.3	21J	Delta	37.96	99.73	1,535	EPI_ISL_7698094	OM146461	SRR18184478
AR-UFRO-0492	34	Male	Alive, not hospitalized	Two doses Sinovac	05-12-21	AY.43.3	21J	Delta	37.91	99.83	1,434	EPI_ISL_7698096	OM146462	SRR18184477
AR-UFRO-0493	56	Female	Alive, not hospitalized	Two doses Sinovac	04-12-21	AY.43.3	21J	Delta	37.56	98.66	1,089	EPI_ISL_7698098	OM146463	SRR18184476
AR-UFRO-0494	27	Male	Alive, not hospitalized	Two doses Sinovac	04-12-21	AY.25.1	21J	Delta	37.91	99.52	1,347	EPI_ISL_7698100	OM146464	SRR18184617
AR-UFRO-0495	34	Female	Alive, not hospitalized	Two doses Sinovac	04-12-21	AY.25.1	21J	Delta	37.66	98.84	1,094	EPI_ISL_7698103	OM146465	SRR18184616
AR-UFRO-0496	18	Female	Alive, not hospitalized	Two doses Sinovac	04-12-21	AY.43.3	21J	Delta	37.96	99.72	1,380	EPI_ISL_7698105	OM146466	SRR18184615
AR-UFRO-0497	33	Male	Alive, not hospitalized	Not vaccinated	04-12-21	AY.122	21J	Delta	37.94	99.73	1,414	EPI_ISL_7698107	OM146467	SRR18184614
AR-UFRO-0499	24	Male	Alive, not hospitalized	Two doses Sinovac	02-12-21	AY.25.1	21J	Delta	37.97	99.8	1,579	EPI_ISL_7698110	OM146468	SRR18184613
AR-UFRO-0500	47	Male	Alive, not hospitalized	Two doses Pfizer	06-12-21	AY.43	21J	Delta	37.75	99.25	968	EPI_ISL_7774637	OM146469	SRR18184612
AR-UFRO-0501	25	Male	Alive, not hospitalized	Not vaccinated	06-12-21	AY.43.3	21J	Delta	37.95	99.8	1,153	EPI_ISL_7698112	OM146470	SRR18184611
AR-UFRO-0502	42	Female	Alive, not hospitalized	Two doses Sinovac	06-12-21	AY.43.3	21J	Delta	36.31	95.38	638	EPI_ISL_7698115	OM146471	SRR18184610

a ID, identifier.

b day-mo-yr.

The availability and increased access to SARS-CoV-2 genomic data will contribute to further studies on the transmission dynamics of the virus, its epidemiology, and the effect of vaccination on the evolution of the virus (10).

### Data availability.

The sequences of the SARS-CoV-2 genomes were deposited in the GISAID and NCBI GenBank (BioProject ID PRJNA810979) databases (see Table 1).
